# RETRACTED: Zanza et al. Ketamine in Acute Brain Injury: Current Opinion Following Cerebral Circulation and Electrical Activity. *Healthcare* 2022, *10*, 566

**DOI:** 10.3390/healthcare14183120

**Published:** 2026-09-21

**Authors:** Christian Zanza, Fabio Piccolella, Fabrizio Racca, Tatsiana Romenskaya, Yaroslava Longhitano, Francesco Franceschi, Gabriele Savioli, Giuseppe Bertozzi, Stefania De Simone, Luigi Cipolloni, Raffaele La Russa

**Affiliations:** 1 Department of Emergency Medicine, Policlinico Gemelli/IRCCS, University of Catholic of Sacred Heart, 00168 Rome, Italy; christian.zanza@live.it (C.Z.); francesco.franceschi@unicatt.it (F.F.); 2 Department of Anesthesia and Critical Care Medicine, Azienda Ospedaliera “SS Antonio e Biagio e C. Arrigo”, 15121 Alessandria, Italy; fpiccolella@ospedale.al.it (F.P.); tatsiana_romenskaya@yahoo.it (T.R.); lon.yaro@gmail.com (Y.L.); 3 Department of Emergency Medicine, Anesthesia and Critical Care Medicine, Michele and Pietro Ferrero Hospital, 12060 Verduno, Italy; 4 Emergency Department, Fondazione IRCCS Policlinico San Matteo, 27100 Pavia, Italy; gabrielesavioli@gmail.com; 5 Department of Clinical-Surgical, Diagnostic and Pediatric Sciences, School in Experimental Medicine, University of Pavia, 27100 Pavia, Italy; 6 Section of Legal Medicine, Department of Clinical and Experimental Medicine, University of Foggia, 71122 Foggia, Italy; giuseppe.bertozzi@unifg.it (G.B.); stefdesim@gmail.com (S.D.S.); raffaele.larussa@unifg.it (R.L.R.)

The journal retracts the article titled “Ketamine in acute brain injury: current opinion following cerebral circulation and electrical activity” [1], cited above.

Following publication, concerns were brought to the publisher’s attention regarding an overlap between this paper [1] and an earlier publication produced by a different authorship group [2].

In accordance with the journal’s standard editorial procedures, the Editorial Office and Editorial Board conducted an investigation, which confirmed an extensive overlap in content with the above-mentioned source. Given the extent of the overlap, the Editorial Board determined that the issue could not be adequately addressed through a correction. The article has therefore been retracted in accordance with MDPI’s retraction policy.

This retraction was approved by the Editor-in-Chief of the journal *Healthcare*.

The authors have been informed of this retraction and have indicated their disagreement with this decision.
